# Supplementary material for: Two orthogonal differentiation gradients locally coordinate fruit morphogenesis
Source: Nat Commun. 2024 Apr 4;15:2912. doi: 10.1038/s41467-024-47325-1 (PMC10995178; doi:10.1038/s41467-024-47325-1)
Supplement: Supplementary file 1 — Supplementary information [file 41467_2024_47325_MOESM1_ESM.pdf]

Supplementary information for

# Two orthogonal differentiation gradients locally coordinate fruit morphogenesis.

Gómez-Felipe A[1]., Branchini E[1]., Wang, B[1]., Marconi M[2,3]., Bertrand-Rakusova H[1]., Stan T[1]., Burkiewicz J[1]., de Folter S[4]., Routier-Kierzkowska A-L[1]., Wabnik K[2,3]., Kierzkowski D.[1]*

[1]Institut de Recherche en Biologie Végétale, Département de Sciences Biologiques, Université de Montréal, 4101 Sherbrooke St E, Montréal, QC, H1X 2B2 Canada

[2]Centro de Biotecnología y Genómica de Plantas (Universidad Politécnica de Madrid (UPM), Instituto Nacional de Investigación y Tecnología Agraria y Alimentaria (INIA, CSIC), Campus de Montegancedo, Pozuelo de Alarcón, 28223 Madrid, Spain

[3]Departamento de Biotecnología-Biología Vegetal, Escuela Técnica Superior de Ingeniería Agronómica, Alimentaria y de Biosistemas, Universidad Politécnica de Madrid (UPM), Madrid 28040, Spain

[4]Unidad de Genómica Avanzada (UGA-LANGEBIO), Centro de Investigación y de Estudios Avanzados del Instituto Politécnico Nacional (CINVESTAV-IPN), CP 36824 Irapuato, Mexico

*Correspondence: daniel.kierzkowski@umontreal.ca

**This file includes:**

Supplementary Figs S1 to S7

# SUPPLEMENTARY FIGURES

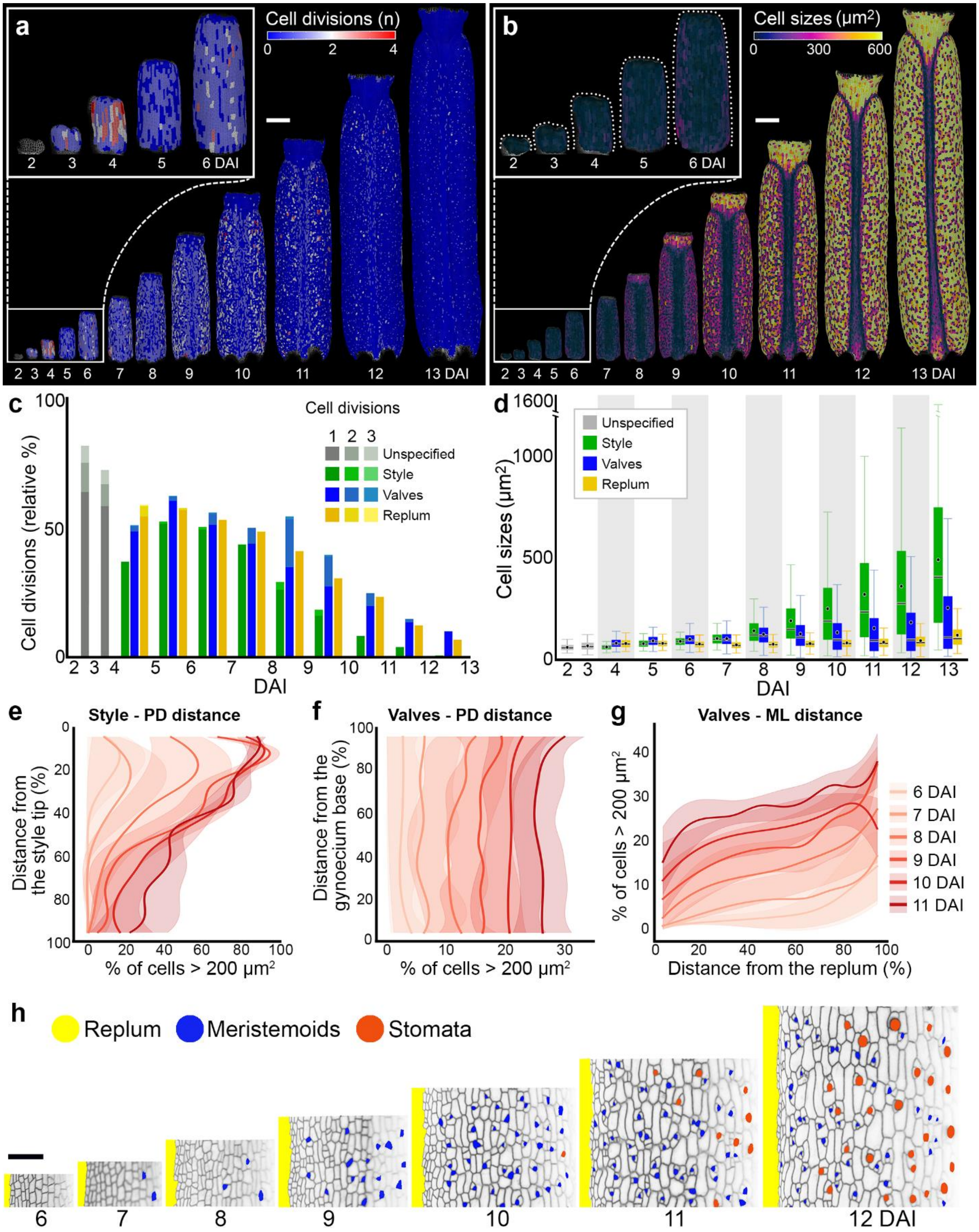

**Supplementary Figure 1. Cellular patterns underlying gynoecium development. a-b,** Heat-maps of cell divisions (a) and cell sizes (b) for the *Arabidopsis thaliana* gynoecium. Dotted lines indicate gynoecium outlines. **c-d,** Quantifications of cell divisions (c) and cell sizes (d) in different regions of the developing gynoecium. (n=136 cells at 3 DAI; n=346 cells at 4 DAI; n=99 (style), 516 (valves), 201 (replum) cells at 5 DAI; n=109 (style), 836 (valves), 347 (replum) cells at 6 DAI; n=190 (style), 1421 (valves), 568 (replum) cells at 7 DAI; n=298 (style), 2459 (valves), 913 (replum) cells at 8 DAI; n=432 (style), 3921 (valves), 1425 (replum) cells at 9 DAI; n=584 (style), 7039 (valves), 2078 (replum) cells at 10 DAI; n=727 (style), 10933 (valves), 2789 (replum) cells at 11 DAI; n=767 (style), valves n=14579 (valves), replum n=3545 (replum) cells at 12 DAI; style n=804 (style), valves n=17027 (valves), replum n=3939 (replum) cells at 13 DAI; three independent time lapse series). The boxplots represent a range between the first and the third quartile and the whiskers include 95% of the values. Lines represent the median and dots represent the mean. **e-g,** Quantifications of cell sizes of style along the proximo distal axis, measured as the distance from the tip (e) and valves along the proximo distal axis measured as distance from the base (f) or along the mediolateral axis measured from the replum (g) (three independent time lapse series). For plots, the distance was normalized, lines represent the average and shaded areas represent standard error (SE). **h,** Tracing of stomata lineages in the valves. Replum in yellow, stomata in orange, meristemoids in blue. DAI indicates days after gynoecium initiation. Scale bars, 100 µm. Related to Figs. 1 and 2.

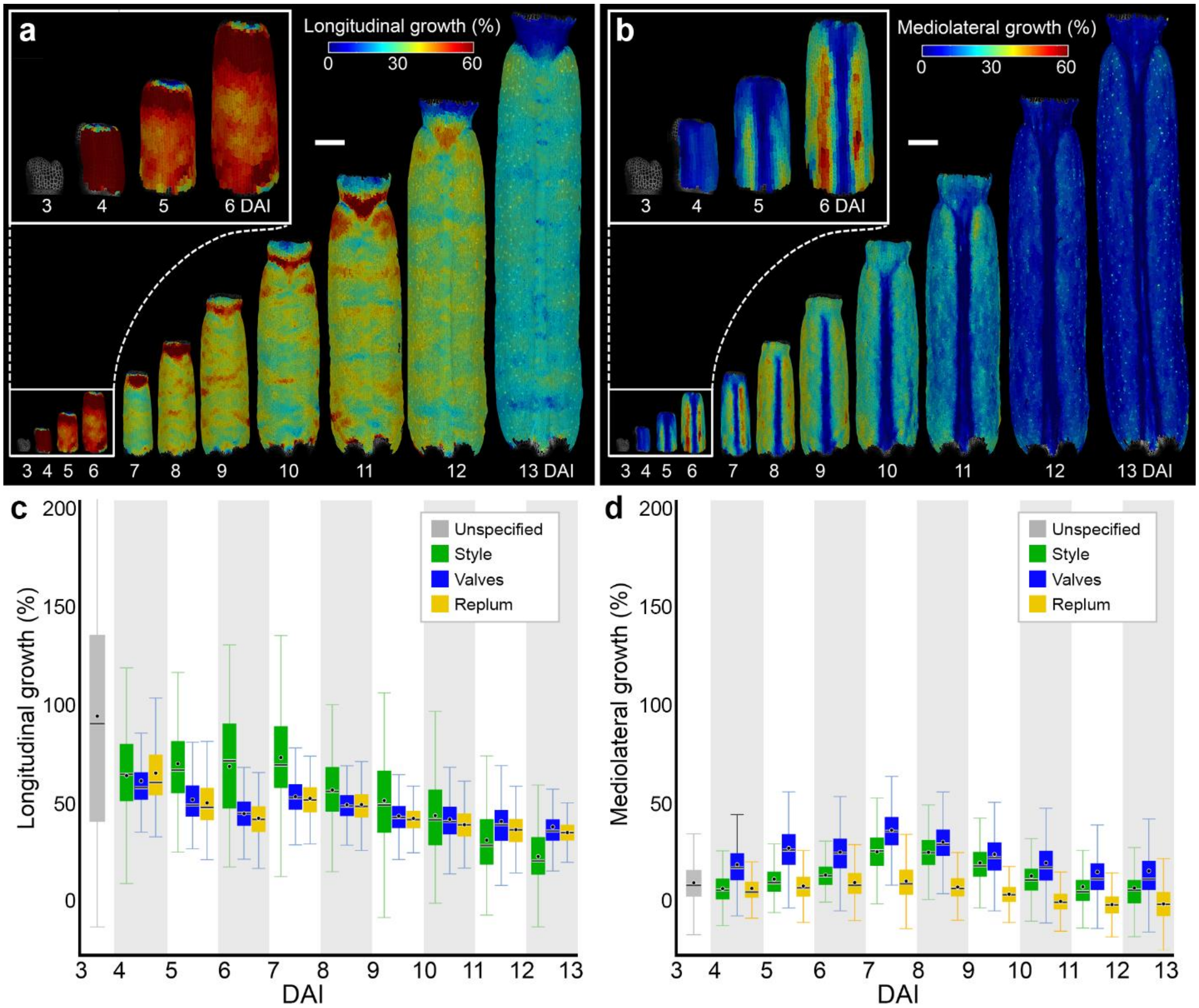


**Supplementary Figure 2. Cellular growth varies along different gynoecium axes. a-b,** Heat-maps of cellular growth along longitudinal (a) and mediolateral (b) axes of the gynoecium in *Arabidopsis thaliana*. **c-d,** Quantification of cellular growth along longitudinal (c) and mediolateral (d) axes of the developing gynoecium. (n=346 cells at 4 DAI; n=99 (style), 516 (valves), 201 (replum) cells at 5 DAI; n=109 (style), 836 (valves), 347 (replum) cells at 6 DAI; n=190 (style), 1421 (valves), 568 (replum) cells at 7 DAI; n=298 (style), 2459 (valves), 913 (replum) cells at 8 DAI; n=432 (style), 3921 (valves), 1425 (replum) cells at 9 DAI; n=584 (style), 7039 (valves), 2078 (replum) cells at 10 DAI; n=727 (style), 10933 (valves), 2789 (replum) cells at 11 DAI; n=767 (style), valves n=14579 (valves), replum n=3545 (replum) cells at 12 DAI; style n=804 (style), valves n=17027 (valves), replum n=3939 (replum) cells at 13 DAI; three independent time lapse series). The boxplots represent a range between the first and the third quartile and the whiskers include 95% of the values. Lines represent the median and dots represent the mean. DAI indicates days after gynoecium initiation. Scale bars, 100 µm. Related to Fig. 2.

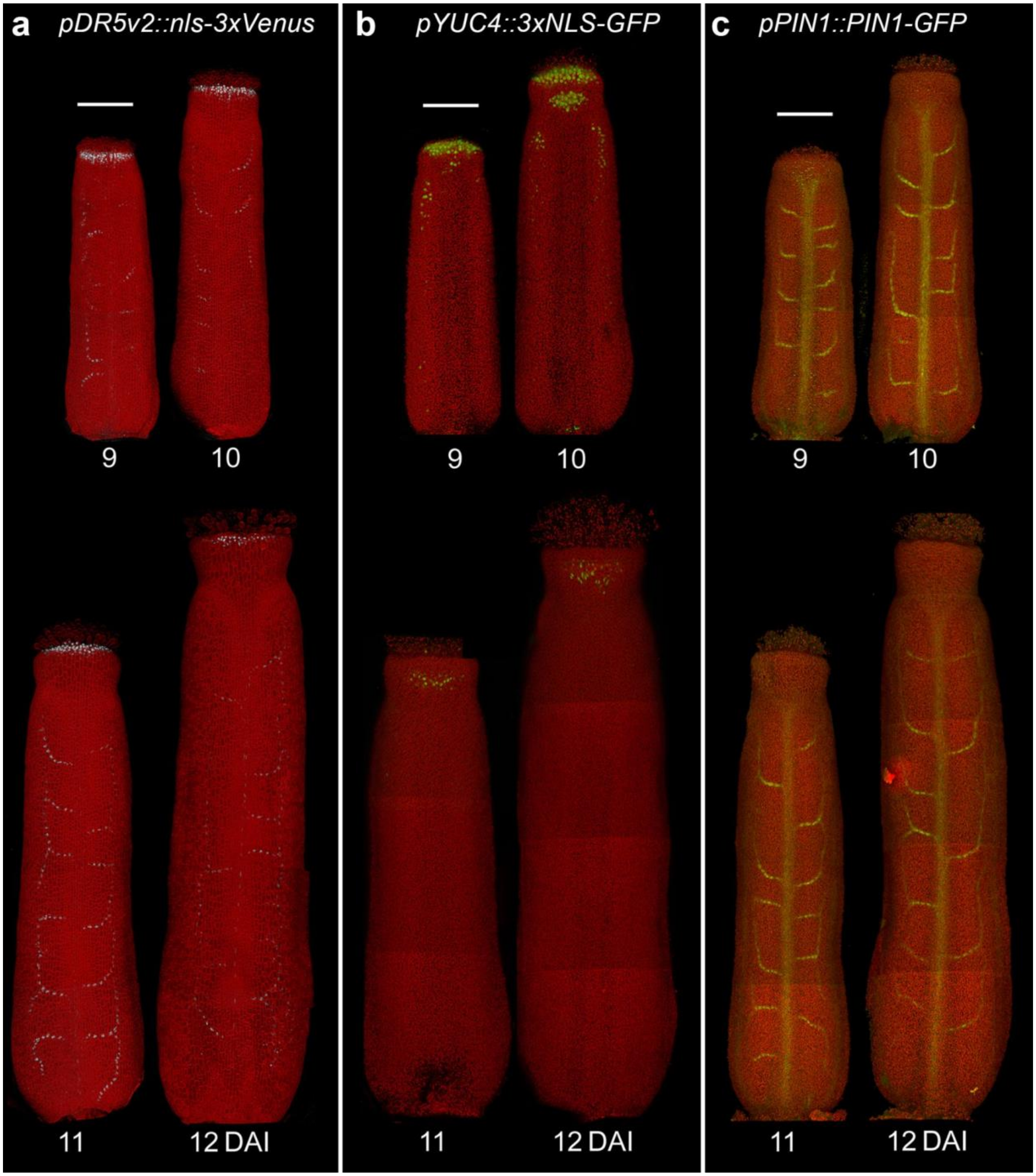


**Supplementary Figure 3. Auxin patterning during gynoecium development. a-c**, Expression patterns of *pDR5v2::nls-3xVenus* (a), *pYUC4::3xNLS-GFP* (b), and *pPIN1::PIN1:GFP* (c) in the gynoecium of *A. thaliana*. DAI indicates days after gynoecium initiation. Scale bars, 100 µm. Related to Fig. 3.

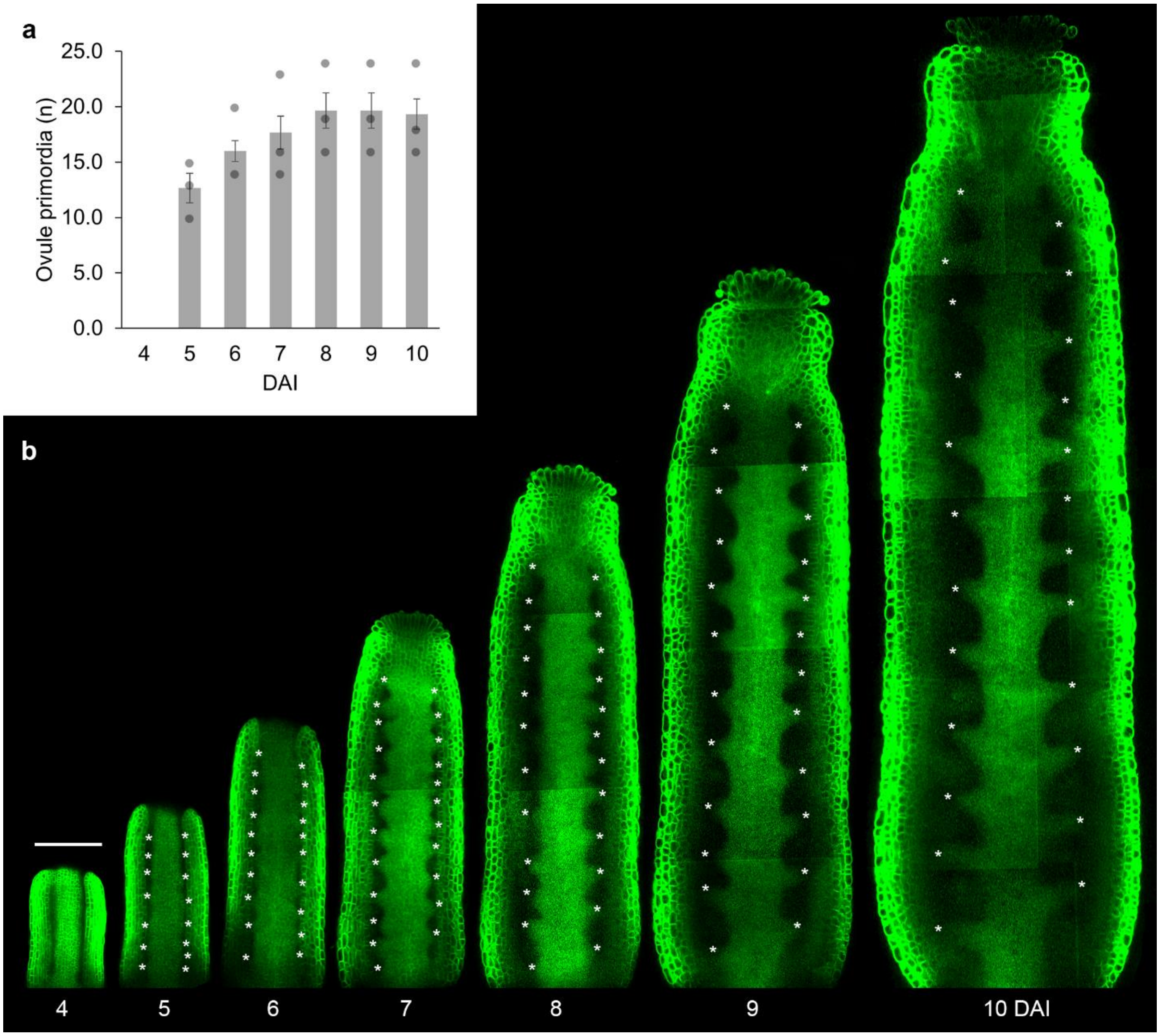


**Supplementary Figure 4. Analysis of the initiation of ovule primordia. a,** Quantification of ovule primordia in the digital longitudinal sections of the gynoecium of *Arabidopsis thaliana*. Error bars indicate SD (three independent time lapse series). **b,** Digital longitudinal sections through the gynoecium. Asterisks mark ovule primordia. DAI: days after gynoecium initiation. Scale bar, 100 µm. Related to Fig. 3.

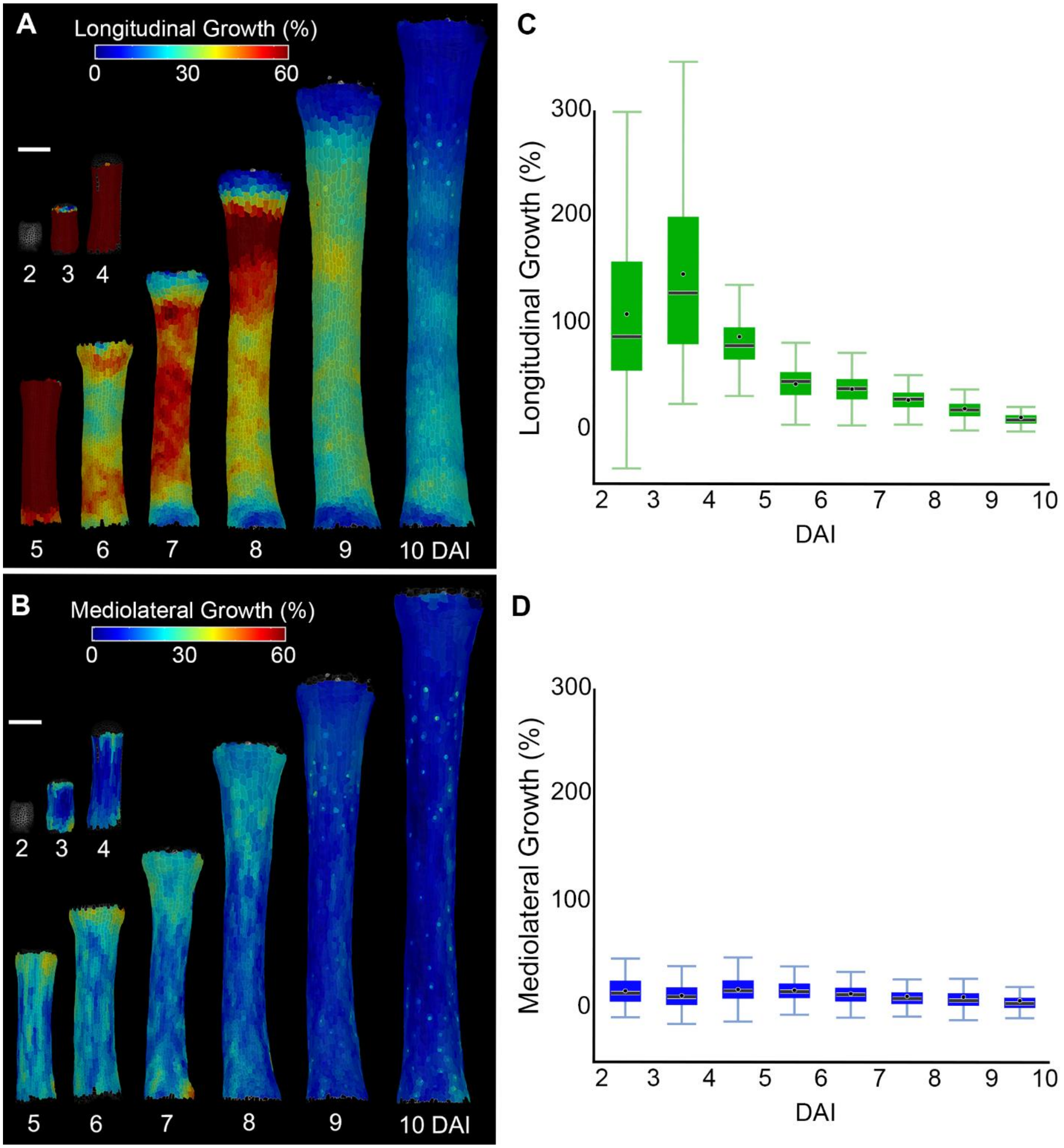


**Supplementary Figure 5. Longitudinal and mediolateral growth in the gynoecium pretreated with NPA. a-b,** Heat maps of longitudinal (a) and mediolateral (b) average growth. **c-d**, Quantification of longitudinal (c) and mediolateral (d) growth in the gynoecia after NPA treatment (n=158 cells at 3DAI; n=166 cells at 4 DAI; n=374 cells at 5 DAI; n=926 cells at 6 DAI; n=1301 cells at 7DAI; n=1531 cells at 8 DAI; n=1540 cells at 9 DAI; n=1575 cells at 10 DAI; three independent time-lapse series). DAI: days after gynoecium initiation. Scale bars, 100 µm. Related to Fig. 4.

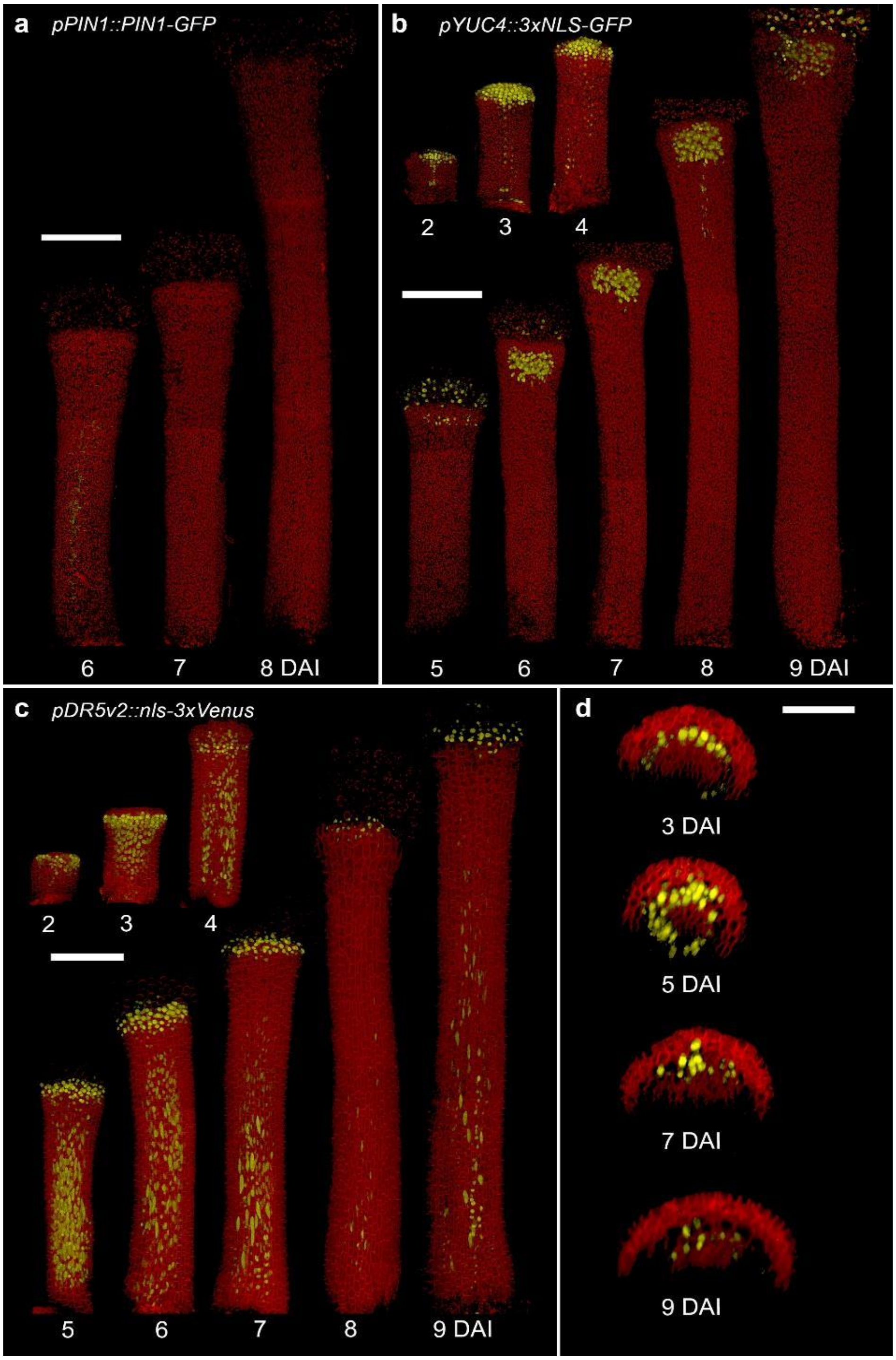


**Supplementary Figure 6. Auxin patterning during the development of valves in the gynoecium pretreated with NPA. a,** Expression patterns of *pPIN1::PIN1:GFP*. **b,** Expression pattern of *pYUC4::3xNLS:GFP*. **c-d,** Expression pattern of *pDR5v2::nls-3xVenus*. DAI: days after gynoecium initiation. Scale bars, 100 µm in (a-c) and 50 µm in (d). Related to Fig. 4.

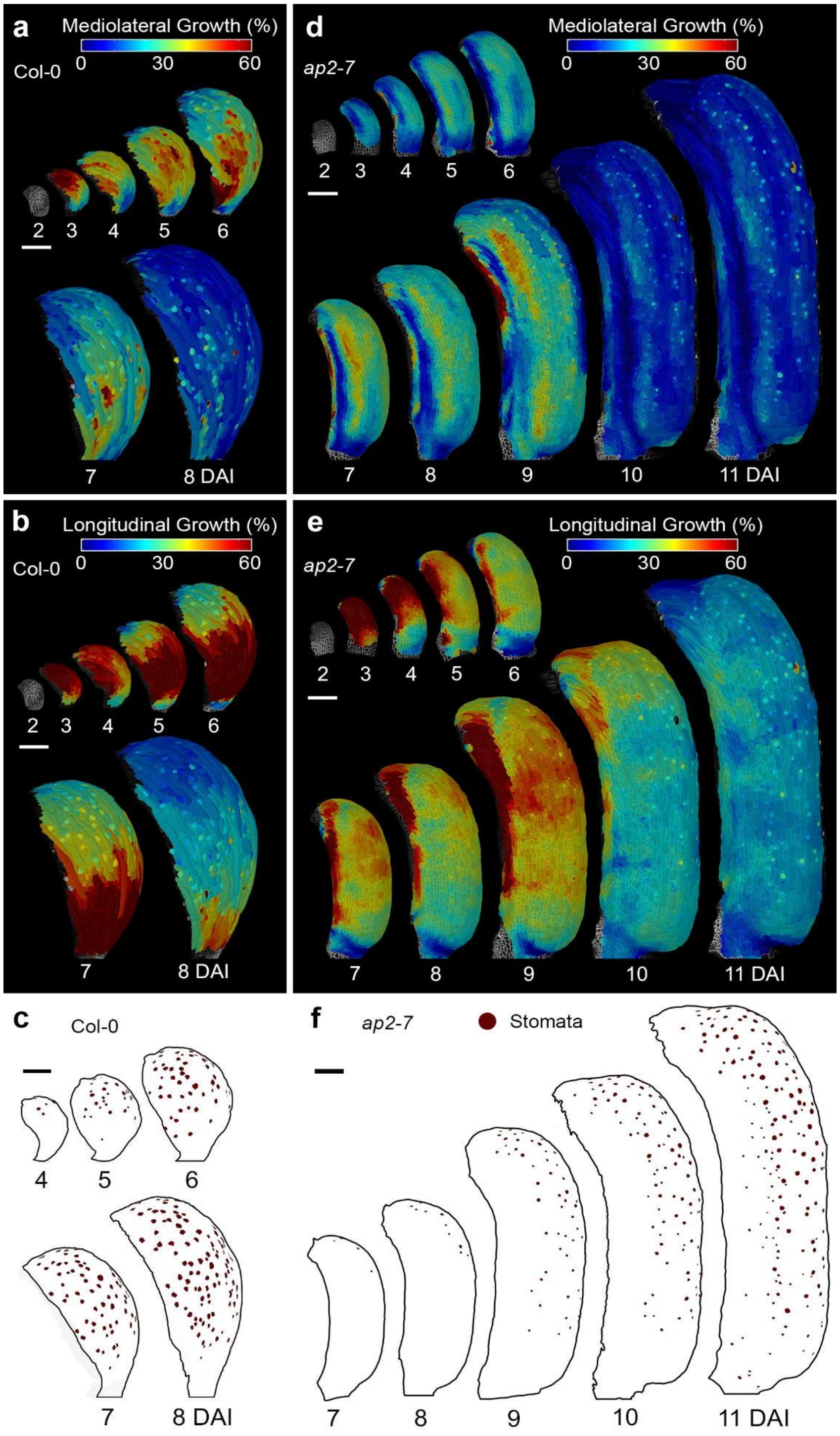


**Supplementary Figure 7. Longitudinal growth, mediolateral growth and stomata distribution in wild-type and *ap2-7* sepals. a-b,** Heat maps of mediolateral (a) and longitudinal (b) average growth in WT sepals. **c**, Stomata distribution in WT sepal. **d-e,** Heat maps of mediolateral (d) and longitudinal (e) average growth in *ap2-7* sepals. **f**, Stomata distribution in *ap2-7* sepal. Three independent time lapse series were acquired for wild-type sepals (n=113-1142 cells) and *ap2-7* sepal (n=44-1478 cells). DAI: days after gynoecium initiation. Scale bars, 100 μm. Related to Fig. 5
