# Supplementary material for: Two orthogonal differentiation gradients locally coordinate fruit morphogenesis
Source: Nat Commun. 2024 Apr 4;15:2912. doi: 10.1038/s41467-024-47325-1 (PMC10995178; doi:10.1038/s41467-024-47325-1)
Supplement: Supplementary file 3 — Description of Additional Supplementary Files [file 41467_2024_47325_MOESM3_ESM.pdf]

Description of Additional Supplementary Files:

Supplementary Movie 1: Area expansion and growth anisotropy in the wild-type gynoecium of Arabidopsis thaliana.

Supplementary Movie 2: Area expansion and cell divisions in the wild-type gynoecium of Arabidopsis thaliana.

Supplementary Movie 3: Longitudinal growth and mediolateral growth in the wildtype gynoecium of Arabidopsis thaliana.

Supplementary Movie 4: Area expansion and cell sizes in the NPA-treated gynoecium of Arabidopsis thaliana.

Supplementary Movie 5: Area expansion in the wild-type and ap2-7 sepals of Arabidopsis thaliana.

Supplementary Movie 6: Area expansion in the crc-1 spt-12 gynoecium of Arabidopsis thaliana.
